# Serodetection of *Ehrlichia canis* amongst dogs in central Namibia

**DOI:** 10.4102/jsava.v86i1.1272

**Published:** 2015-06-01

**Authors:** Rutendo Manyarara, Ulf Tubbesing, Minty Soni, Bruce H. Noden

**Affiliations:** 1 Rhino Park Veterinary Clinic, Windhoek, Namibia; 2 Department of Health Sciences, Polytechnic of Namibia, Namibia

## Abstract

*Ehrlichia canis* is a major pathogen in dogs throughout Africa, yet it has not been reported in Namibia. The aim of this study was to determine the seroprevalence of canine ehrlichiosis in central Namibia using the ImmunoComb assay (Biogal, Galed Laboratories). The study included 76 dogs that presented to the Rhino Park Veterinary Clinic in the north-western suburb of Khomasdal, Windhoek, Namibia, as well as 30 stray dogs from the Windhoek branch of the Society for the Prevention of Cruelty to Animals. Of the 106 dogs tested, 53.8% were seropositive at titres > 1:80. Dogs that presented with symptoms of *E. canis* infection had a significantly higher seroprevalence (86.6%) compared with apparently healthy dogs (41.6%) (*P* = 0.00). Location of habitation was significant (*P <* 0.017), with a high percentage of dogs exposed to *E. canis* living in the northern or north-western part of Windhoek. As the first study to serologically establish *E. canis* as a major pathogen in dogs in central Namibia, it is notable that the highest proportion of seropositive dogs came from low-income areas. Further investigation is necessary to describe the ecology of this important tick-borne pathogen of companion animals in Namibia.

## Introduction

Canine ehrlichiosis is caused by *Ehrlichia canis*, a rickettsial Gram-negative species in the family Anaplasmataceae (Dumler *et al*. 2007). Throughout the world, *E. canis* is transmitted by the brown dog tick, *Rhipicephalus sanguineus* (Harrus & Waner 2011; Nicholson *et al*. 2010), and is one of four species in the genus (*E. canis*, *Ehrlichia chaffeensis*, *Ehrlichia ewingii* and *Ehrlichia ruminantium*) that cause serious disease in animals and humans (Dumler *et al*. 2007; Louw, Allsopp & Meyer 2005; Murphy *et al*. 1998). *Ehrlichia canis* infection in dogs has been reported widely in Africa (Kamani *et al*. 2013; Kelly, Eoghain & Raoult 2004; Matthewman *et al*. 1993; Pretorius & Kelly 1998; Socolovschi *et al*. 2012), but not in Namibia specifically. Therefore, the aim of this study was to determine the seroprevalence of canine ehrlichiosis in central Namibia using a point-of-care assay.

## Materials and methods

The study was conducted at the Rhino Park Veterinary Clinic in Khomasdal, a north-western suburb of Windhoek, Namibia. Data were collected over a period of 8 weeks from dogs that presented to the clinic as well as from stray dogs under the care of the Windhoek branch of the Society for the Prevention of Cruelty to Animals (SPCA). In all cases, participation was voluntary and depended on ease of handling the patient and written informed consent from the patient's direct owner or guardian. The study protocol was approved by the Namibian Ministry of Agriculture, Water and Forestry.

At the clinic, veterinarians evaluated dogs for presenting with symptoms such as inappetence, lethargy and emaciation. Blood samples (2 mL – 5 mL) were obtained from central sites (e.g. the jugular vein) by venipuncture. After initial evaluation, these dogs were assigned to either of two groups, (1) those suspected of *E. canis* infection or (2) those with non-specific symptoms or that came to the clinic for elective procedures. At inclusion in the study, the sex and age of the dog were noted. Clinic veterinarians also evaluated 30 stray dogs from the local SPCA during the same period to provide another estimate of the potential prevalence of the disease in central Namibia.

The ImmunoComb kit (Biogal, Galed Laboratories, Israel), a solid-phase dot enzyme-linked immunosorbent assay (ELISA) based on a crude antigen of *E. canis* (Israeli strain), was used according to the manufacturer's instructions to detect IgG-specific antibodies. The manufacturer's cut-off value for positive samples was a titre ≥ 1:80. Reported sensitivity and specificity of this test are 96% and 87%, respectively, when compared with the ‘gold standard’ immunofluorescent antibody test for Nigerian strains of *E. canis* (Okewole & Adejinmi 2009). Similarly, sensitivity and specificity for Middle Eastern strains of *E. canis* are reported at 86% and 98%, respectively, with this ELISA (Harrus *et al*. 2002). Serum samples were assayed immediately after processing and the remainder of each sample was stored at -20 °C for further analysis.

Data were analysed using the PASW Statistics 18 (SPSS Inc, Chicago, IL) software package. Chi-square and Fisher exact tests were used to compare proportions between groups and *t*-tests and Analysis of variance (ANOVA) were used for comparisons of continuous variables. Age of animals was analysed in three groups: < 1 year, between 1 and 8 years, and > 8 years. A *P*-value of less than 0.05 was considered statistically significant for all tests.

## Results

A total of 106 dogs from various areas of Windhoek and the surrounding Khomas region were evaluated. Of these, 76 were dogs that visited the clinic and 30 were apparently healthy strays from the local SPCA. *Ehrlichia canis* infection was suspected for the majority (86.2%) of the clinic-evaluated dogs as they presented with inappetence, lethargy and emaciation. The majority of dogs sampled were males (53.5%) and younger than 8 years (92.7%) (Table 1). Ticks were found on 17 (22.4%) of the clinic-evaluated dogs, with 13 (76.5%) having clinical signs as opposed to 4 (23.5%) without clinical signs.

**TABLE 1:** Seroprevalence of *Ehrlichia canis* infection amongst a sample of dogs (*n* = 106) in central Namibia.

		Dogs evaluated		Seropositive dogs	
Categories	Variable	*n*	Proportion of sample	*n*	Prevalence in test group (**%**)
Sex^a^	Male	53	0.54	28	52.8
	Female	46	0.46	27	58.7
Age^b^	Young (1 year)	35	0.51	22	62.9
	Adult (1–8 years)	29	0.42	15	51.7
	Old (> 8 years)	5	0.07	3	60.0
Symptom status	Clinic; positive	46	0.44	40*	86.6
	Clinic; negative	30	0.28	13	42.3
	SPCA; negative	30	0.28	12	40.0
Source and geographic area: Pets	West^c^	12	0.11	5	41.7
	North^d^	28	0.26	21	75.0
	East^e^	7	0.07	2	28.6
	South^f^	8	0.08	3	37.5
	Outside of town^g^	4	0.04	2	50.0
	Not registered	17	0.16	12	70.6
Source and geographic area: SPCA	–	30	0.28	12	40.0

SPCA, Society for the Prevention of Cruelty to Animals.

^a^, Sex of 7 SPCA dogs was not recorded; ^b^, Ages of seven clinic dogs were not recorded nor was age estimated for SPCA dogs; ^c^, Windhoek West, Rocky Crest; ^d^, Khomasdal, Katatura; ^e^, Avis, Ludwigsdorf, Klein Windhoek, Eros; ^f^, Pioneers Park, Olympia, Klein Kuppe; ^g^, Otjiwarongo, Gobabis.

*, *P* ≤ 0.05

Of the 106 dogs tested, 57 (53.8%) tested seropositive for antibodies against *E. canis* at titres > 1:80. Dogs with symptoms of *E. canis* had a higher seroprevalence (86.6%) than dogs without symptoms (42.3%) or stray dogs (40.0%) (*P* = 0.00). Seroprevalence was similar for male and female dogs and did not differ significantly between the age groups (Table 1). Location of habitation was significant, with a high proportion of seropositive dogs coming from the northern or north-western parts of Windhoek (Khomasdal and Katatura areas) (Table 1). Ticks were found on more dogs from the northern or north-western areas (32.1%) than on dogs from other areas (15.4%), but the difference was not significant (*P* < 0.150).

## Discussion

This is the first serological study to establish *E. canis* as a major pathogen in dogs in central Namibia. The high seroprevalence (53.8%) of *E. canis* in the sample of Namibian dogs is comparable to results from studies in rural Zimbabwe (34%) (Kelly *et al*. 2004) and urban South Africa (42%) (Pretorius & Kelly 1998). The highest proportion of seropositive dogs came from the lower-income areas of Khomasdal and Katatura, which reflects a similar trend to that reported in Bloemfontein, South Africa (Pretorius & Kelly 1998). The only described vector of canine ehrlichiosis, *Rhipicephalus sanguineus* (the brown dog tick), is the principal tick species found on dogs throughout central and southern Namibia (Matthee *et al*. 2010). With a higher prevalence of ticks found on dogs from lower-income areas, this pattern may be due to a lack of access to tick control (Pretorius & Kelly 1998).

## Conclusion

Although the involvement of only one veterinary clinic and the limited timescale and sample size may introduce several limitations, this study demonstrates that dogs in central Namibia are frequently exposed to *Ehrlichia* species, the most likely being *E. canis*. Based on the serological detection, further molecular investigation of Namibian dogs and their ticks is required to verify the presence of *Ehrlichia* species.
